# Hybrid Copolymerization of Ethylene Oxide and *tert*-Butyl Methacrylate with Organocatalyst

**DOI:** 10.3390/polym13152546

**Published:** 2021-07-31

**Authors:** Wenhao Xiao, Liguo Xu, Pan Liu, Yang Chen, Jie Zhang, Jinbao Xu

**Affiliations:** 1 Guangdong Provincial Key Laboratory of Functional Soft Condensed Matter, School of Materials and Energy, Guangdong University of Technology, Guangzhou 510006, China; xiaowenhao1232021@163.com (W.X.); pangliu1232021@163.com (P.L.); 1112002017@mail2.gdut.edu.cn (Y.C.); zhangjiez2019@163.com (J.Z.); 2 School of Materials Science and Engineering, South China University of Technology, Guangzhou 510640, China; xuliguojkkl@163.com

**Keywords:** ethylene oxide, *tert*-butyl methacrylate, hybrid copolymerization, solid polymer electrolyte

## Abstract

Hybrid copolymerization of structurally different, reactivity and mechanism distinct monomers (e.g., cyclic and vinyl type monomers) is of great interest and challenge for both academic research and practical application. Herein, ethylene oxide-*co*-*tert*-butyl methacrylate-*co*-poly(ethylene glycol) benzyl methacrylate (EO-*co*-BMA-*co*-*b*PEO), a statistical copolymer was synthesized via hybrid copolymerization of EO and BMA using an uncharged, non-nucleophilic organobase *t*-BuP_4_ as the catalyst. Detailed characterizations indicate that hybrid copolymerization of ethylene oxide and vinyl monomer forms a statistical copolymer concurrently with the transesterification of *tert*-butyl group and oligomer PEO anions. The application of the copolymer as all solid lithium-ion battery polymer electrolyte was investigated by detecting the ionic conductivity (σ) with electrical impedance spectrum measurement.

## 1. Introduction

Living (co)polymerization is a powerful tool to construct well-defined homopolymers or copolymers with a narrow dispersity, high molecular weight, controlled composition and architecture (e.g., block, brush and star polymers). Therefore, the development of efficient copolymerization strategies is of great interest from both the academic and industrial perspective. Particularly, copolymers are commonly prepared by sequentially adding different monomers or using a mixture of co-monomers [1,2,3,4,5,6]. Combining polymerization techniques with different mechanisms is an alternative route to fabricate copolymers from conventional methods, but it is still a great challenge in terms of polymer synthesis. Among them, concurrent vinyl-addition and ring-opening hybrid copolymerization is of particular importance because large quantities of (meth)acrylates and oxiranes have been widely used to prepare various functional copolymers [7,8,9,10]. Despite its potential applications, only a few studies have reported such copolymerization reactions concerning vinyl and cyclic monomers, including cyclic imino ethers, dioxaphospholanes, aziridines and (meth)acrylates [11,12,13,14], methacrylate (MMA)/*tert*-butyl methacrylate (BMA) and *ε*-caprolactone (CL) [15,16], and vinyl ethers and oxiranes [17,18,19], and the reaction mechanism is zwitterionic, anionic or cationic. 

Regarding this hybrid copolymerization, the combination of ethylene oxide (EO) and vinyl monomers is attractive to us as PEO-based polymers could be used as solid polymer electrolytes (SPE), which have been proved to greatly improve the safety and processability of lithium-ion batteries [20,21,22,23,24]. Although the anionic copolymerization of EO and vinyl monomers (e.g., MMA, BMA) have been reported in previous publications, the structure of the resulting products were indeterminate. The authors were unable to confirm whether the two monomers underwent hybrid copolymerization because their analyses were based on the crude products without any purification [25,26,27]. 

According to Fetter’s classification of monomer reactivity, the difficulty for anionic copolymerization of vinyl monomers and oxiranes was due largely to the facts that a vinyl group cannot nucleophilically attack a stable oxonium ion as well as the reactivity differences of monomers [28]. Although BMA and EO are reactive anionic species, the successful block polymerization of two monomers is known to depend critically on the relative reactivity of comonomers, i.e., on the relative stability of the conjugated anions. It has previously been reported that poly (alkyl methacrylate) anions could initiate the polymerization of ethylene oxide, polyethylene glycol methacrylate and alkyl methacrylate [29]. Thus, it is possible to develop a catalyst that could activate both ends from a vinyl monomer and EO and proceed the subsequent copolymerization without formation covalent growing ends. Actually, we demonstrated the successful copolymerization of MMA/BMA and CL [15,16]. Each monomer could form propagating species during the copolymerization to facilitate the further combination of either similar or dissimilar monomers, forming a statistical copolymer with the help of super base *t*-BuP_4_. Having this in mind, we studied the hybrid copolymerization of EO and BMA, and investigated the application of the obtained products as lithium-ion battery SPE in the present work.

## 2. Materials and Methods

### 2.1. Materials

Ethylene oxide (EO) from Aladdin was dried over calcium hydride (CaH_2_) and distilled under argon atmosphere prior to use. *tert*-Butyl methacrylate (BMA) from Aldrich were freshly distilled under vacuum and stored under an argon atmosphere. Benzyl alcohol (BnOH) from Aldrich (Shanghai, China) were dried over sodium with nitrogen protective atmosphere and distilled under vacuum after refluxing four hours. Tetrahydrofuran (THF) from Sinopharm (Shanghai, China) were freshly distilled from sodium/benzophenone and stored under an argon atmosphere. *t*-BuP_4_ from Aldrich was used as received and other reagents from Sinopharm or Aldrich were used as received.

### 2.2. Characterizations

Proton nuclear magnetic resonance (^1^H NMR) and carbon 13 nuclear magnetic resonance (^13^C NMR) were recorded on a Bruker AV400 NMR spectrometer (Rheinstetten, Germany) by using deuterated chloroform (CDCl_3_) as the solvent and tetramethylsilane (TMS) as the internal standard. Fourier transform infrared (FTIR) spectra were recorded on a Thermo Scientific Is50R FT-IR spectrometer (Thermo Scientific, Waltham, MA, USA). Samples were thoroughly mixed with potassium bromide (KBr) and pressed into a pellet for measurement. The spectra were collected at 64 scans with a spectral resolution of 4 cm^−1^. The number average molecular weight (*M*_n_) and dispersity (*Ð*) were measured at 35 °C on a Waters size exclusion chromatography (SEC, Milford, DE, USA) equipped with a model Waters 1515 pump, a differential refractive index detector model 410 (RI). A series of monodisperse poly(ethylene oxide) were used as the standard and THF was used as the eluent at a flow rate of 1.0 mL/min. Sample fractions were conducted on LC98II high performance liquid chromatograph equipment (Thermo Scientific, USA) with a P98II pump, a UV98II type UV visible detector (wavelength: 190~700 nm) and a UC-3265 fraction collector. THF was used as the eluent at a flow rate of 1.0 mL/min at 35 °C. Thermogravimetric analysis (TGA) was performed on a TA SDT Q600 instrument (METTLER, Greifensee, Switzerland) under nitrogen atmosphere at a heating rate of 10 °C/min in the range from 25 °C to 600 °C. Differential scanning calorimetry (DSC) was conducted on a Mettler Toledo DCS3 (METTLER, Switzerland) under a nitrogen flow of 50 mL/min. Samples were quickly heated to 150 °C and kept for 10 min to remove thermal history and then cooled to −75 °C at a rate of 10 °C/min. Finally, they were reheated to 150 °C at the same rate. The glass transition temperature (*T*_g_) was taken as that centered at the transition. The ionic conductivity (σ) of the polymer electrolytes were measured by using ac impedance spectroscopic technique with Bio-Logic Science Instrument VMP3B-10 workstation (Bio-Logic, Seyssinet-Pariset, France). The polymeric membranes were sandwiched between two stainless-steel electrodes in an “SS/SPE/SS” configuration and encapsulated in a coin cell. Before measuring, the cell was first heated at 60 °C for 2 h allowing the electrolyte to contact well with electrodes. The ac impedance spectra were recorded over a temperature range from 25 °C to 95 °C in the frequency range from 0.1 to 106 Hz. The values of σ were calculated according to the equation: σ = l/(SR), where l is the thickness of membranes, S is the area of stainless-steel electrode, and R is the bulk resistance of the SPE membrane.

### 2.3. Hybrid Copolymerization of EO and BMA

A typical copolymerization was performed as follows. EO (0.53 g, 12 mmol, 120 equiv.), BMA (1.70 g, 12 mmol, 120 equiv.), BnOH (3.50 μL, 0.1 mmol, 1.0 equiv.), and THF (6.0 mL) were placed in a flamed and argon purged round-bottom flask equipped with a magnetic stirrer at −40 °C. *t*-BuP_4_ (63 μL, 0.05 mmol, 0.8 M in hexane) was added under argon with a syringe to start the polymerization after three freeze-pump-thaw cycles of the above solution. The polymerization was kept at −40 °C for 2 h and completed with another 46 h at 45 °C by adding a few drops of acetic acid. The product was dissolved in THF and precipitated into a large excess of ether. White solid powder was obtained after drying under vacuum overnight. The homopolymerization of EO and copolymerization of EO and BMA under other reaction conditions was carried out with a similar procedure. The copolymers were abbreviated as BMA_n_-*co*-EO-*co*-*b*PEO_m_, n denotes the molar ratio of BMA components and m refers to the molar ratio of transesterification resulting parts in the copolymers, which were calculated from ^1^H NMR analysis.

### 2.4. Homopolymerization of BMA

A typical reaction was performed as follows. BMA (1.70 g, 12 mmol, 120 equiv.), BnOH (3.50 μL, 0.1 mmol, 1.0 equiv.) and THF (3.50 mL) were placed in a flamed and argon purged round-bottom flask equipped with a magnetic stirrer. *t*-BuP_4_ (63 μL, 0.05 mmol, 0.8 M in hexane) was added under argon with a syringe to start the polymerization after three freeze-pump-thaw cycles of the above solution. A few drops of acetic acid were added to stop the reaction after 6 h and white solid powder was obtained by precipitating the mixture into a large excess of hexane.

## 3. Results and Discussion

**Figure 1:**
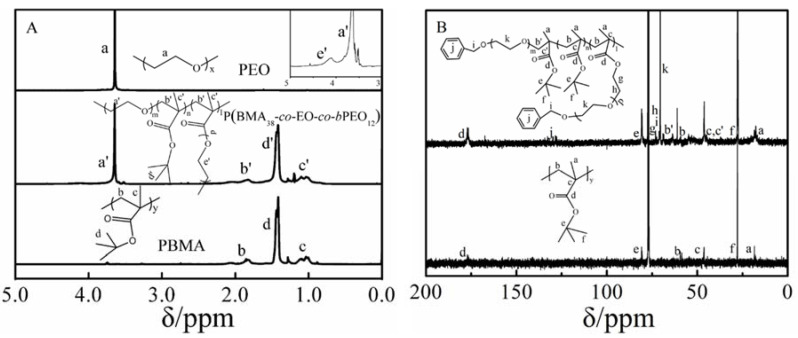
(**A**) ^1^H NMR spectra of PEO, PBMA and their copolymers, (**B**) ^13^C NMR spectra of PBMA and P(BMA_38_-*co*-EO-*co*-*b*PEO_12_).

As reported before, hybrid copolymerization makes cyclic and vinyl monomers copolymerize into a statistical copolymer possibly, where each monomer can form propagating species and combine with either similar or dissimilar monomers. In the present work, we first proceeded the homopolymerization of PEO and PBMA using *t*-BuP_4_ as the catalyst, benzyl alcohol as the initiator and THF as the solvent. The polymerization was in a controlled manner with narrow molecular weight dispersities (*Ð*) (Table 1). Then, the copolymerization of EO and BMA was carried out from −40 °C (2 h) to 40 °C (46 h) and the obtained products showed that the copolymerization was initiated from benzyl alcohol (Figure S1, BMA_38_-*co*-EO-*co*-*b*PEO_12_). The ^1^H NMR analysis of the resulting product showed that the proton signals for PEO and PBMA were all presented in the product while a new peak appeared at 4.10 ppm (Figure 1A), indicating that EO and BMA segments were both in the obtained product but with something new. The proportions of each peaks were calculated from the integration of signals and we found that the theoretical integration areas ratio of peak b’(S_b’_, Figure 1A) and d’ (S_d’_, Figure 1A) should have been 1/4.5 while the calculated value was 1/3.94, indicating that ~12% mol *tert*-butyl groups of BMA were replaced. In addition, size exclusion chromatography (SEC) with refractive index detector showed multimodal and a much broader *Ð* of the product compared to the previous reported homopolymerization or copolymerization with *t*-BuP_4_ organocatalyst (Table 1) [30,31,32].

**Scheme 1:**
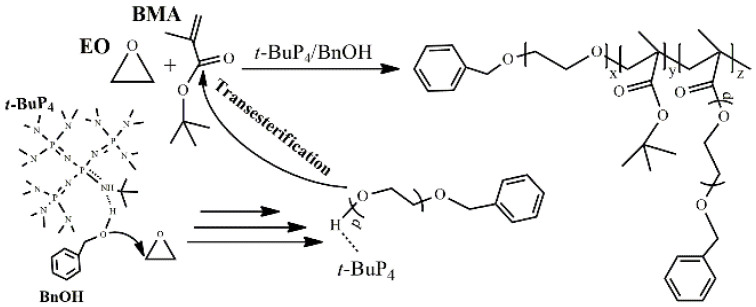
Hybrid copolymerization of EO and BMA.

The existence of the high and low molecular weight part, broadening of *Ð* was also found when synthesizing poly(methyl methacrylate)-*b*-poly(ethylene oxide) copolymers and it was attributed to the transesterification of the pendant ester groups of poly(methyl methacrylate) and the alkoxide derivative of PEO [33]. The transesterification reaction has also been used to prepare poly(methyl methacrylate-*g*-ethylene oxide) graft copolymers [34]. Having this in mind, the transesterification reaction may occur between oligomers of PEO and *tert*-butoxy groups of PBMA part of the copolymer. However, the transesterification reaction may also occur between the BMA monomer and the PEO oligomers active chain end, a new monomer which could possibly be copolymerized with BMA or EO formed in this way. Extra experiments were promoted to confirm the transesterification reaction. One was the ring opening polymerization of EO which was performed with *t*-BuP_4_ as the catalyst and BnOH as the initiator in the presence of PBMA (reaction condition was just as the copolymerization of EO and BMA); there was no transesterification observed (Figure S2). Another one was that the aliquot was withdrawn from the EO and the BMA copolymerization reaction mixture and a new monomer formed (Figure S3). Thus, we deduce a new peak and the broadening of *Ð* from the transesterification reaction between the oligomer PEO active chain end and a *tert*-butoxy group of BMA monomer (Scheme 1). The copolymers were abbreviated as BMA_n_-*co*-EO-*co*-*b*PEO_m_, where n denotes the molar ratio of BMA components and m refers to the molar ratio of transesterification resulting parts in the copolymers, which were calculated from the ^1^H NMR analysis.

**Figure 2:**
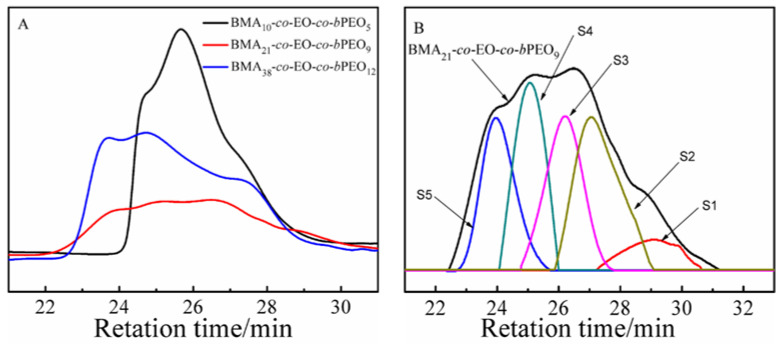
(**A**) SEC curves of copolymers, (**B**) SEC curves of BMA_21_-*co*-EO-*co*-*b*PEO_9_ fractionations.

In order to further study the copolymerization process, we carried out the polymerization with various monomer feeds (Table 1). The *Ð* of the obtained product increased with the BMA content increasing and for all the samples about 5–16% *tert*-butyl groups of BMA were replaced. Although ^1^H NMR analyses indicated the existence of PEO and PBMA in all the samples, we still could not conclude that the obtained products were the copolymers of EO and BMA because of the relative broad *Ð* of the samples (Figure 2A). In order to confirm the copolymerization of EO and BMA, the fractionation of the obtained product with a preparation gel permeation chromatography was proceeded. The molar ratio of BMA units to EO calculated from the ^1^H NMR measurement and the number average molecular weight (*M*_n_) calculated from the SEC of each fractionation are listed in Table 2. There was no significant difference between each fractionation and the unfractionated copolymer (Figure S4) while the *Ð* value of each fractionation was relatively narrow and unimodal (Figure 2B). The SEC and ^1^H NMR results based on fractionation suggest that the product was a statistical copolymer, otherwise it was not the homopolymer blend of PEO and PBMA.

The ^13^C NMR measurement was conducted to further confirm the statistical copolymerization of EO and BMA, the obtained product exhibited all the carbon signals of PEO and PBMA (Figure 1B). The peaks at 46.6 ppm due to the quaternary carbon (−CH_2_C(CH_3_)−) of BMA split into two peaks and the new peaks appeared at 68.8 ppm (−CH_2_C(CH_3_)−) due to the connection of EO and BMA indicating that the obtained product was the statistical copolymer of EO and BMA. The new peaks at 73.1 and 70.5 ppm were assigned to ethylene carbons (−CH_2_C(CH_3_)COOCH_2_CH_2_−) also confirming the transesterification of oligomer PEO with the *tert*-butyl group of BMA.

Figure S5 (ESI†) shows that differential scanning calorimetry (DSC) curves of the copolymers exhibited only one glass transition temperature (*T*_g_) and a melting peak appeared with a higher EO content, presumably due to the crystallization of EO segments. It was noted that the *T*_g_ values did not obey the FOX equation for statistical copolymers as the transesterification segments having a similar structure to poly(ethylene glycol) methyl methacrylate (*T*_g_ ~ −70 °C ), causing the *T*_g_ to just increase a little with the BMA content increasing [35,36]. The DSC curves of the copolymers also displayed a further reduction in the crystallinity of the PEO segments which was found to be inversely proportionate to the BMA molar content. The melting endotherm specific to PEO disappeared when the EO content decreased to 50% as long BMA chain prevented the alignment and nucleation of the PEO parts, further indicating the statistical incorporation of BMA and EO units. DSC curves of fractionations also showed only one glass transition as the pre-fractionation copolymer (Figure S6).

**Scheme 2:**
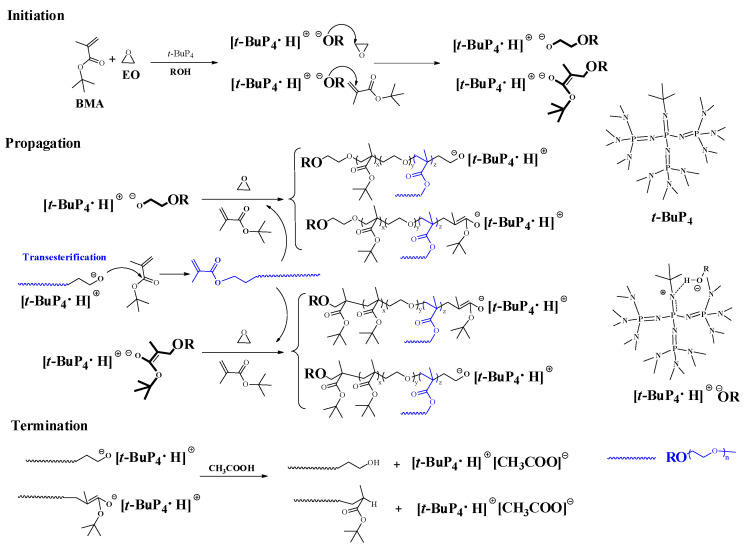
A plausible mechanism for *t*-BuP_4_ catalyzed hybrid copolymerization of EO and BMA.

We could now conclude that the hybrid copolymerization of EO and BMA formed a poly(ethylene oxide-*co*-*tert*-butyl methacrylate-*co*-benzyl poly(ethylene oxide) methacrylate) (BMA-*co*-EO-*co*-*b*PEO) statistical copolymer concurrently with the transesterification of the *tert*-butoxy group and oligomer PEO anionic active chain end with *t*-BuP_4_ as the catalyst. Based on the above results, a possible mechanism for the EO/BMA hybrid copolymerization was proposed (Scheme 2). The initiator was first activated via H-bonding between the *t*-BuP_4_ and hydroxyl group of the initiator to generate an alkoxide and the attack of the alkoxide on the methylene carbon of EO or double bond carbon of BMA produced two kinds of active centers, which makes the hybrid copolymerization possible. Then, the long active chain centers were newly formed by the continuous regenerated reaction of BMA or EO, leading to two active centers. Moreover, the new formed monomer from the transesterification also joined the propagation process. Finally, the propagating chain was deactivated by the addition of acetic acid. Scheme 2 just describes some aspects of the reaction mechanism and the copolymerization mechanism seems much more complex. More studies (e.g., reaction kinetics, reaction component interactions and computational modeling) could promote our understanding of this new kind hybrid copolymerization, however, which is beyond the scope of the present manuscript and will be the subject of future work.

**Figure 3:**
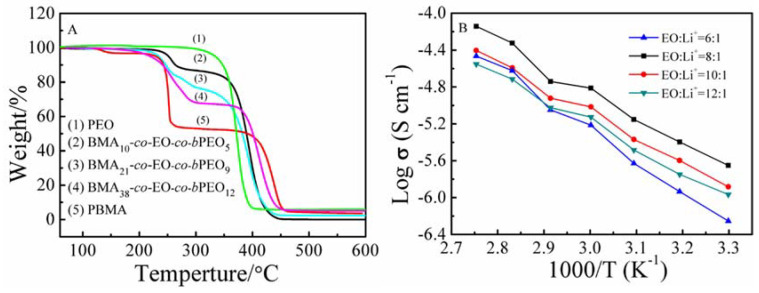
(**A**) TGA curves of PEO, PBMA and their copolymer products, (**B**) ionic conductivity with different EO:Li^+^ of BMA_38_-*co*-EO-*co*-*b*PEO_12_.

We finally focused on the application of PEO-based SPE because of its several specific advantages such as high safety, easy fabrication, low cost and excellent compatibility with lithium salts, which makes it the most widely studied candidate in all solid states of secondary lithium batteries. However, the linear PEO usually has a high crystallinity which can restrain the ionic transition especially at lower temperature as the lithium ions often translate in the amorphous region [37,38]. Researchers have explored different approaches to reduce the crystallinity in order to improve the ionic conductivity of PEO-based electrolytes, including blending, modifying and making PEO derivatives [39,40,41,42]. Hybrid copolymerization of EO and vinyl monomers allows for the synthesis of PEO-based amorphous materials conveniently. To evaluate the thermal stability of the novel PEO-based SPE, thermogravimetric analysis (TGA) was conducted. As shown in Figure 3B, the copolymers showed almost no weight loss until 240 °C which can meet the practical requirement of lithium ionic batteries and are much safer than liquid electrolytes [43]. Moreover, the two-stage weight loss also confirmed the copolymerization of EO and BMA, the first weight loss increased with the BMA components increasing.

Then, we detected the ionic conductivity (σ) of this novel SPE as being one of the most critical properties for lithium-ion batteries. According to previous publications, each Li ion could coordinate with approximately five EO units. Thus, a range of EO to Li ion ratios were conducted to investigate the σ of amorphous BMA_38_-*co*-EO-*co*-*b*PEO_12_ lithium SPE at different temperature. As shown in Figure 3B, the σ increased with the temperature increase and the room temperature σ was ~2.23 × 10^−6^ S/cm, which was much higher than that of the PEO homopolymer [20]. When the temperature increased to 60 °C, the σ was 1.54 × 10^−5^ S/cm with a molar ratio of EO:Li^+^ = 8:1. Furthermore, the statistical copolymer could be converted to a single-ion SPE via a lithiation reaction with carboxyl, which could be formed through the hydrolysis of the *tert*-butyl group of PBMA. The electrochemical performance of this novel SPE was investigated in depth in our laboratory.

## 4. Conclusions

In summary, the hybrid copolymerization of EO and BMA was realized by the use of an uncharged Brönsted strong base *t*-BuP_4_ for the first time. Our results have clearly confirmed that the different two monomers could initiate each other forming statistical copolymers without homopolymerization and the transesterification between the reactive PEO oligomer and the *tert*-butoxy group of BMA was coexistence with the hybrid copolymerization. Thus, the structural diversity of the PEO-based statistical copolymer could be readily enriched by the use of vinyl monomers carrying different substituents and initiators with a different functional group. The hybrid copolymerization of EO and BMA offered a simple synthetic route to prepare amorphous PEO-based SPE, which was difficult to prepare by other means. Ionic conductivity results indicated that this novel SPE may be used in lithium-ion batteries.

## Figures and Tables

**Table 1:** Characterization data of BMA and EO (co)polymerization product ^a^.

Samples	*f* ^b^	*M*_n_ × 10^−4^ (g/mol) ^c^	*Ð* ^c^	*F*_EO_^d^ (mol %)
PBMA	100/0	1.38	1.26	0
PEO	0/250	0.98	1.19	100
BMA_10_-*co*-EO-*co*-*b*PEO_5_	30/170	1.45	1.55	85
BMA_21_-*co*-EO-*co*-*b*PEO_9_	70/130	1.83	1.78	70
BMA_38_-*co*-EO-*co*-*b*PEO_12_	100/100	2.01	1.92	50
BMA_49_-*co*-EO-*co*-*b*PEO_16_	130/70	2.14	2.26	35
BMA_59_-*co*-EO-*co*-*b*PEO_16_	140/60	2.25	2.32	25

^a^ BnOH was used as initiator and solvent was THF; ^b^ molar ratio of BMA to EO in feed; ^c^ obtained from SEC; ^d^ molar fraction of BMA units in copolymer detected by ^1^H NMR.

**Table 2:** Characterization data of fractionations for BMA_21_-*co*-EO-*co*-*b*PEO_9_.

Samples	*T*_m_ ^a^	*M*_n_ × 10^−4^ (g/mol) ^b^	*Ð* ^b^	*F*_EO_ (mol %) ^c^
S1	42.4	0.20	1.25	10.0
S2	44.2	0.62	1.14	9.2
S3	44.6	1.14	1.12	9.3
S4	44.8	1.98	1.18	9.2
S5	45.0	3.69	1.13	9.4

^a^ Determined by DSC; ^b^ obtained from SEC; ^c^ molar fraction of BMA units in copolymer detected by ^1^H NMR.

## Data Availability

Not applicable.

## Supplementary Materials

The following are available online at https://www.mdpi.com/article/10.3390/polym13152546/s1, Figure S1: ^1^H NMR spectrum of the EO and BMA hybrid copolymerization product (BMA_38_-*co*-EO-*co*-*b*PEO_12_), Figure S2: ^1^H NMR spectrum for *t*-BuP_4_ catalyzed ROP of EO with in the presence of PBMA (*M*_n,SEC_ = 1.25 × 10^4^ g/mol, *Ð* = 1.23 ) (BnOH was initiator and THF was solvent, reaction temperature was from −40 °C (2 h) to 40 °C (46 h), the *M*_n,SEC_ of the obtained PEO is about 4.0 × 10^3^ g/mol with *Ð* = 1.19), Figure S3: ^1^H NMR spectrum of aliquot for *t*-BuP_4_ catalyzed ROP of EO and BMA after 4 h (BnOH was initiator and THF was solvent, reaction temperature was from −40 °C (2 h) to 40 °C (46 h)), Figure S4: ^1^H NMR spectra of EO and BMA copolymerization product (BMA_21_-*co*-EO-*co*-*b*PEO_9_) fractionations, Figure S5: DSC curves of PEO, PBMA and their copolymer products, Figure S6: DSC curves of BMA_21_-*co*-EO-*co*-*b*PEO_9_ fractionations.
