# Supplementary material for: Hybrid Copolymerization of Ethylene Oxide and tert-Butyl Methacrylate with Organocatalyst
Source: Polymers (Basel). 2021 Jul 31;13(15):2546. doi: 10.3390/polym13152546 (PMC8347643; doi:10.3390/polym13152546)
Supplement: Supplementary file 1 [file polymers-13-02546-s001.zip › polymers-1277582-supplementary.pdf]

Supporting Information

Hybrid Copolymerization of Ethylene Oxide and *tert*-Butyl Methacrylate with Organocatalyst

Wenhao Xiao,[1] ‡ Liguo Xu,[2] ‡ Pan Liu,[1] Yang Chen,[1] Jie Zhang,[1] Jinbao Xu[1,*]

[1] Guangdong Provincial Key Laboratory of Functional Soft Condensed Matter, School of Materials and Energy, Guangdong University of Technology, Guangzhou 510006, China;

[2] School of Materials Science and Engineering, South China University of Technology, Guangzhou 510640, China

* Correspondence: xujinbao@gdut.edu.cn (Jinbao Xu)

‡ These authors contributed equally to this work.

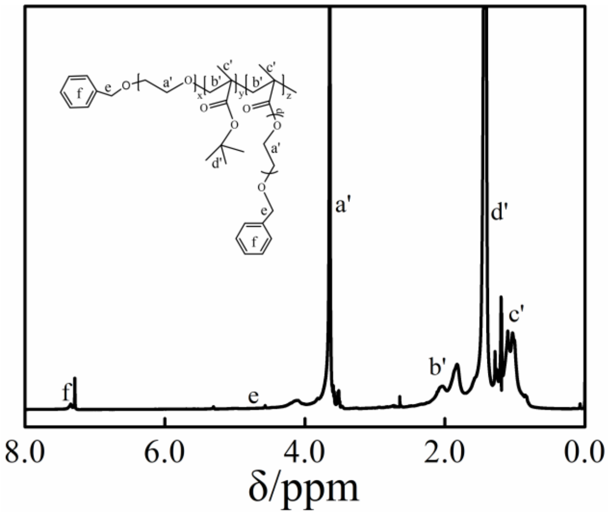

Figure S1. ^{1}H NMR spectrum of the EO and BMA hybrid copolymerization product (BMA$_{38}$-*co*-EO-*co*-*b*PEO$_{12}$).

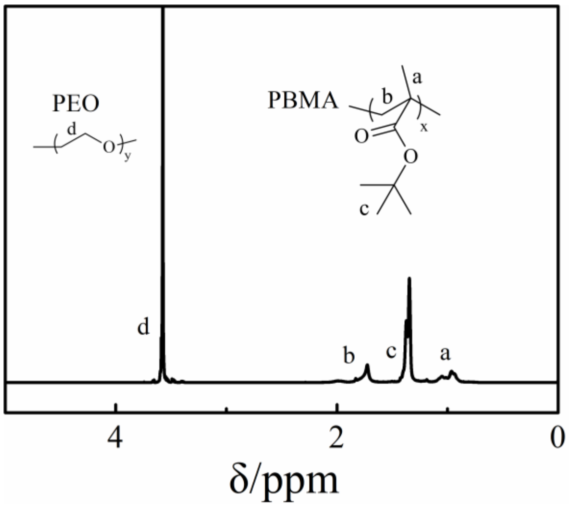

Figure S2. ^{1}H NMR spectrum for *t*-BuP$_4$ catalyzed ROP of EO with in the presence of PBMA ($M_{n,SEC}$ = 1.25×10^4 g/mol, $Đ$ = 1.23) (BnOH was initiator and THF was solvent, reaction temperature was from - 40 °C (2 h) to 40 °C (46 h), the $M_{n,SEC}$ of the obtained PEO is about 4.0×10^3 g/mol with $Đ$ = 1.19).

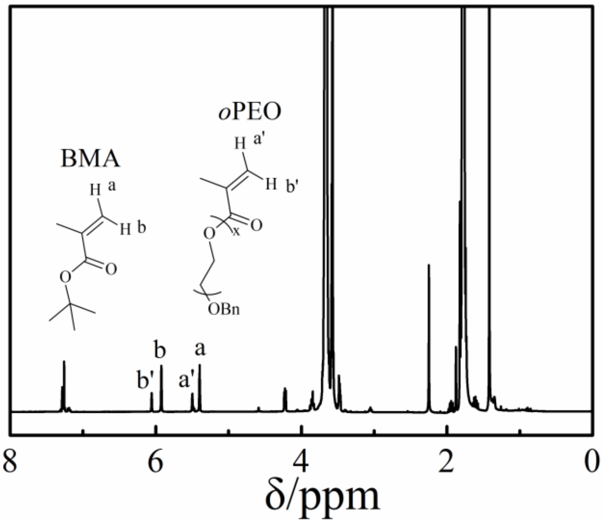

Figure S3. ^{1}H NMR spectrum of aliquot for *t*-BuP$_4$ catalyzed ROP of EO and BMA after 4 h (BnOH was initiator and THF was solvent, reaction temperature was from - 40 °C (2 h) to 40 °C (46 h)).

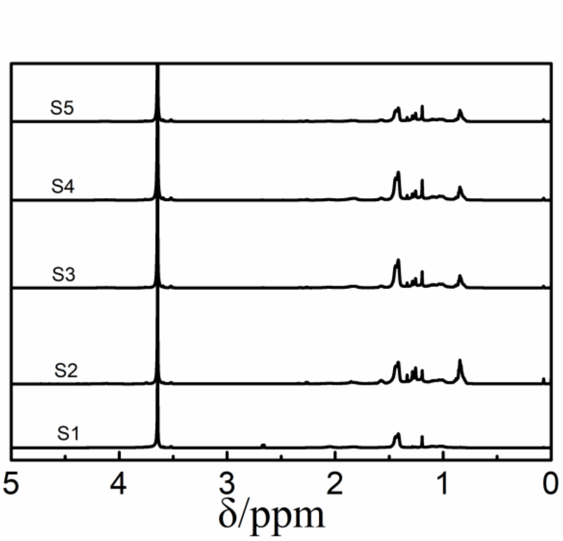

Figure S4. ^{1}H NMR spectra of EO and BMA copolymerization product (BMA$_{21}$-*co*-EO-*co*-*b*PEO$_9$) fractionations.

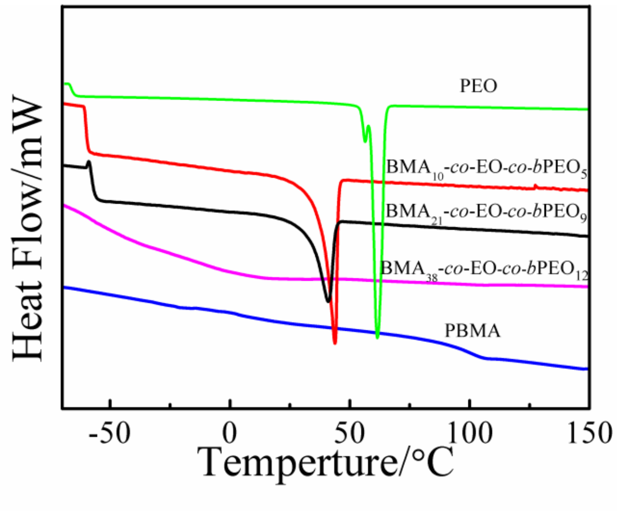

Figure S5. DSC curves of PEO, PBMA and their copolymer products.

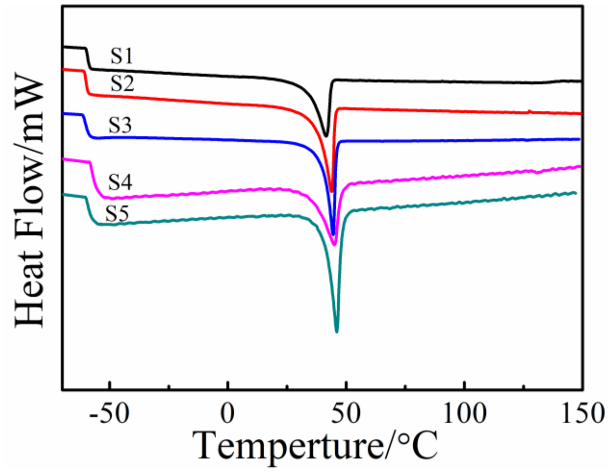

Figure S6. DSC curves of BMA_{21}-*co*-EO-*co*-*b*PEO_9 fractionations.
